# Supplementary material for: Immunofluorescent Localization of Plakoglobin Is Altered in Endomyocardial Biopsy Samples from Dogs with Clinically Relevant Arrhythmogenic Right Ventricular Cardiomyopathy (ARVC)
Source: Vet Sci. 2021 Oct 23;8(11):248. doi: 10.3390/vetsci8110248 (PMC8623220; doi:10.3390/vetsci8110248)
Supplement: Supplementary file 1 [file vetsci-08-00248-s001.zip › vetsci-1409170-supplementary.pdf]

Supplemental

Table S1

Case #	Breed	Sex	Age (Y)	ECG	Holter	Echo	Histo	Confocal	Striatin	Group
1	Cane Corso	F	7	VT	NA	WNL	Fibro	PG-ID	NA	non-ARVC
2	Labrador	M	3	3 AVB	NA	WNL	Normal	PG-ID	NA	non-ARVC
3	Labrador	M	6	3 AVB	NA	DCM	Fibro/Lymphocytes	PG-ID	NA	non-ARVC
4	Labrador	F	9	3 AVB	NA	WNL	Normal	ND	NA	non-ARVC
5	Dobermann	F	7	VT	NA	WNL	Fibro	PG-ID	NA	non-ARVC
6	Labrador	M	3	AF	NA	DCM	Fibro/Lymphocytes	PG-ID	NA	non-ARVC
7	Labrador	M	4	3 AVB	NA	WNL	Fibro	PG-ID	NA	non-ARVC
8	Bulldog	M	1	AF	NA	DCM	Fibro	PG-ID	NA	non-ARVC
9	Mongrel	M	1	SR	NA	DCM	Fibro/Lymphocytes	PG-ID	NA	non-ARVC
10	Boxer	M	3	3 AVB	NA	WNL	Normal	PG-ID	NA	non-ARVC
**11**	**Boxer**	**M**	**1**	**VT**	**NA**	**DCM**	**Fibro / fat**	**PG-MIS**	**NA**	**clin-ARVC**
12	German Shepherd	M	4	AFIB+ 3 AVB	NA	DCM	Normal	PG-ID	NA	non-ARVC
13	Argentinian D.	F	11	VT	NA	PE	Normal	PG-ID	NA	non-ARVC
14	Beagle	M	10	VPCs	NA	DCM	Fibro	ND	NA	non-ARVC
15	Dogue de Bordeaux	F	6	3 AVB	NA	DCM	Lymphocytes	PG-ID	NA	non-ARVC
16	Napolitain Mastiff	FS	10	SVT	NA	PE	Fibro	PG-ID	NA	non-ARVC
17	Great Dane	M	6	3 AVB	NA	DCM	Normal	PG-ID	NA	non-ARVC
18	Mongrel	F	5	SR	NA	DCM	Lymphocytes	PG-ID	NA	non-ARVC
19	Mongrel	M	5	SR	NA	DCM	Fibro	PG-ID	NA	non-ARVC
20	Labrador	M	5	3 AVB	NA	WNL	Lymphocytes	PG-ID	NA	non-ARVC
**21**	**Boxer**	**F**	**2**	**VT**	**NA**	**DCM**	**Fibro / fat**	**PG-MIS**	**NA**	**clin-ARVC**
**22***	**Mongrel**	**M**	**11**	**VT**	**NA**	**CMVD**	**Lymphocytes**	**PG-MIS**	**NA**	**non-ARVC**
23	Dogue de Bordeaux	F	8	AFL	NA	DCM	Fibro/Lymphocytes	PG-ID	NA	non-ARVC
24	French Bulldog	M	8	SR	NA	DCM	Fibro/Lymphocytes	PG-ID	NA	non-ARVC
25	Dogue de Bordeaux	M	9	CVP	NA	PE	Fibro	PG-ID	NA	non-ARVC
26	Weimaraner	M	7	AF	NA	DCM	Fibro	PG-ID	NA	non-ARVC
27	Mongrel	M	6	VPCs - TB	NA	DCM	Fibro	PG-ID	NA	non-ARVC
28	Dogue de Bordeaux	M	4	AF	NA	DCM	Lymphocytes	PG-ID	NA	non-ARVC
29	Labrador	M	1	AF	NA	DCM	Fibro/Lymphocytes	ND	NA	non-ARVC
30	Dogue de Bordeaux	M	2.6	AF	NA	WNL	Fibro	PG-ID	NA	non-ARVC
31	Boxer	FS	2	FAT	NA	DCM	Fibro	PG-ID	NA	non-ARVC
32	Broholmer	M	2	AF	NA	WNL	Fibro	PG-ID	NA	non-ARVC
33	Golden Retriever	M	0.5	FAT	NA	DCM	Fibro	PG-ID	NA	non-ARVC
34	Labrador	M	4	VPCs	NA	DCM	Fibro	PG-ID	NA	non-ARVC
35	Mongrel	M	10	VT	NA	DCM	Fibro	PG-ID	NA	non-ARVC
36	Bull Mastiff	M	6	AF	NA	DCM	Fibro	PG-ID	NA	non-ARVC
37	Rhodesian Ridgeback	M	4	AF	NA	DCM	Fibro	ND	NA	non-ARVC
38	German Shepherd	M	8	SR	NA	DCM	Fibro/Lymphocytes	PG-ID	NA	non-ARVC
39	Dobermann	M	2.5	FAT	NA	DCM	Fibro	PG-ID	NA	non-ARVC
40	Golden Retriever	FS	7	AF	NA	DCM	Fibro	PG-ID	NA	non-ARVC
41	Dobermann	M	2	SR	NA	DCM	Fibro/Lymphocytes	PG-ID	NA	non-ARVC
42	Labrador	M	0.6	SR	NA	DCM	Fibro	ND	NA	non-ARVC
**43**	**English Bulldog**	**M**	**9**	**VT**	**NA**	**ARVC**	**Fibro / fat**	**PG-MIS**	**NA**	**clin-ARVC**
44	Boxer	M	9	SR	765 VE	WNL	Fibro / fat	PG-ID	NEG	occ-ARVC
45	Boxer	F	6	SR	11 VE	WNL	Fat	PG-ID	UNKN	occ-ARVC
46	Boxer	M	6	SR	280 VE	WNL	Fibro / fat	PG-ID	NEG	occ-ARVC
47	Boxer	F	5	SR	4 VE	WNL	Fat	PG-ID	NEG	non-ARVC
48	Boxer	F	7	SR	3 VE, 9 SVE	WNL	Fibro / fat	PG-ID	UNKN	occ-ARVC
49	Boxer	F	6	SR	4400 VE	WNL	Fibro / fat	PG-ID	UNKN	occ-ARVC

Supplemental Table S1: Patient information including breed, sex, age, ECG and Holter findings, echocardiographic findings (Echo), results of EMB histopathology (Histo), results of immunofluorescence microscopy (Confocal), striatin status, when available, and grouping. Shaded boxes denote dogs with a histopathologic diagnosis of ARVC. Bold text indicates dogs with mislocalized plakoglobin (**PG-MIS**). Dog #22 (asterisk) had PG-MIS, despite a diagnosis of non-ARVC cardiac disease. Abbreviations: AF (atrial fibrillation), ARVC (arrhythmogenic right ventricular cardiomyopathy, AVB (atrio-ventricular block), Clin-ARVC (ARVC with clinical signs), (CMVD (chronic mitral valve disease), DCM (dilated cardiomyopathy phenotype), FAT (focal atrial tachycardia), LP (lymphoplasmacytic), NEG (negative), ND (non-diagnostic), Non-ARVC (non-ARVC cardiac disease), Occ-ARVC (Occult ARVC, no clinical signs), PE (pericardial effusion), PC (cadherin), PG (plakoglobin), PG-ID (plakoglobin localized to intercalated disc), PG-MIS (plakoglobin mislocalized), SR (sinus rhythm), SVE (supraventricular ectopy), SVT (supraventricular tachycardia), VE (ventricular ectopy), VT (Ventricular tachycardia), WNL (within normal limits), UNKN (unknown).
